# Immobilization of azide-functionalized proteins to micro- and nanoparticles directly from cell lysate

**DOI:** 10.1007/s00604-023-06068-4

**Published:** 2023-12-22

**Authors:** Gunjan Saini, Mrugesh Krishna Parasa, Katherine N. Clayton, Julia G. Fraseur, Scott C. Bolton, Kevin P. Lin, Steven T. Wereley, Tamara L. Kinzer-Ursem

**Affiliations:** 1 Weldon School of Biomedical Engineering, Purdue University, West Lafayette, IN 47906 USA; 2 School of Mechanical Engineering, Purdue University, West Lafayette, IN 47906 USA; 3 Department of Biochemistry, Purdue University, West Lafayette, IN 47906 USA

**Keywords:** Click chemistry, Gold nanoparticles, Magnetic beads, Protein labeling, Calmodulin, Calcineurin, Protein kinase A

## Abstract

Immobilization of proteins and enzymes on solid supports has been utilized in a variety of applications, from improved protein stability on supported catalysts in industrial processes to fabrication of biosensors, biochips, and microdevices. A critical requirement for these applications is facile yet stable covalent conjugation between the immobilized and fully active protein and the solid support to produce stable, highly bio-active conjugates. Here, we report functionalization of solid surfaces (gold nanoparticles and magnetic beads) with bio-active proteins using site-specific and biorthogonal labeling and azide-alkyne cycloaddition, a click chemistry. Specifically, we recombinantly express and selectively label calcium-dependent proteins, calmodulin and calcineurin, and cAMP-dependent protein kinase A (PKA) with N-terminal azide-tags for efficient conjugation to nanoparticles and magnetic beads. We successfully immobilized the proteins on to the solid supports directly from the cell lysate with click chemistry, forgoing the step of purification. This approach is optimized to yield low particle aggregation and high levels of protein activity post-conjugation. The entire process enables streamlined workflows for bioconjugation and highly active conjugated proteins.

**Graphical Abstract:**

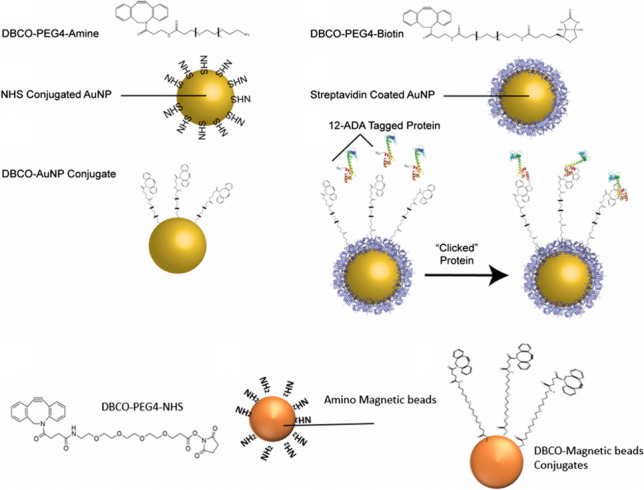

**Supplementary Information:**

The online version contains supplementary material available at 10.1007/s00604-023-06068-4.

## Introduction

As the field of bionanotechnology is rapidly expanding, researchers are taking advantage of novel bioconjugation methods to produce stable, highly bio-active micro- and nanoparticle protein conjugates. Gold nanoparticles are widely used in nanoscience for a variety of applications due to many advantageous properties, including biocompatibility, unique resonance properties, ease of manufacturing, and commercial availability [1, 2]. Likewise, magnetic particles are widely used in various bioscience applications as they can be easily manipulated, isolated from solutions, and magnetic particle in a variety of conformations, sizes, and surface functionalization are commercially available [3]. Advances in bioconjugation have been applied across a number of fields, including supported catalysts, fabrication of biosensors [4, 5], vehicles for nanomedicine [6–9] imaging contrast agents [10–14], and multiplexed assays of protein concentration, interactions, and activity [15, 16]. In biomolecular sensing, researchers take advantage of the large surface area-to-volume ratio of micro- and nanoparticles to detect low concentrations of target analytes [17]. These bionanosensors often have biomolecules conjugated to the nanoparticle surface to serve as the binding and detection element. Likewise, various supports (e.g., glass slides, paper, silicon, plastic beads, magnetic beads) were used for attaching proteins for production of functional materials. Forming biomolecule–particle conjugates allows for the production of hybrid materials with characteristics that can be used in multiple aspects, such as heating or plasmonic properties in conjunction with biological targeting [18, 19] [20].

Proteins are commonly immobilized onto nanoparticle surfaces and magnetic beads [21] [22] via non-specific adsorption [23], affinity binding [24], or with covalent chemistries, such as primary amine [25, 26], thiol [27], or maleimide [28, 29] bonds. Non-specific adsorption and affinity conjugation are attractive approaches; the methods are relatively straight-forward to implement, and many examples exist [30]. However, drawbacks to non-specific absorption include low protein activity and the dissociation from the particle in the presence of complex sample matrices [31–33]. In contrast, popular covalent chemistries offer a permanent bond between the protein and particle. However, these chemistries can cause poor particle stability, leading to aggregation, and proteins are often conjugated to the surface heterogeneously via a number of possible conjugations sites on the protein surface, which can lead to a loss of overall specific activity [34]. Further, covalent conjugation often requires prior purification of the protein, which significantly adds to workflow complexity and cost. The ability to specifically engineer proteins in recombinant expression systems and conjugate these proteins to particles directly out of complex cell lysate is an attractive approach [35, 36]. Developing methods to conjugate proteins to nanoparticles or magnetic beads directly from cell expression systems would save the time and cost associated with purification procedures that are employed in current laboratory and industrial workflows and would aid in high-throughput nanoparticle design and manufacturing. Furthermore, implementation of high-throughput protein conjugation methods in conjugation with protein assay platforms might be used for quick and reliable study of function of proteins in a cost-effective manner.

There have been many recent advances in chemoenzymatic protein labeling to exploit selective tagging for bioconjugation applications [37]. Such approaches allow for labeling proteins in complex mixtures and streamlined laboratory workflows for site-specific tagging of enzymes onto substrates. N-myristoylation has been used to site-specifically label both natural substrate proteins [38, 39] and engineered proteins co-translationally in *E. coli* with 12-azidododecanoic acid (12-ADA) [35, 36, 40]. N-myristoyl transferase (NMT) covalently appends 12-ADA to the N-terminus of substrate proteins carrying a peptide recognition sequence, and no other *E. coli* proteins receive the tag. Given the bio-orthogonality of the azide functional group on 12-ADA, the substrate protein can then be conjugated to alkyne-functionalized fluorophores or surfaces directly from cell lysate [36, 40]. This method was used to engineer calmodulin (CaM), an important Ca^2+^ sensing protein, to accept the 12-ADA tag with no loss in protein function [35, 41]. Calcineurin (CaN), a Ca^2+^/CaM-dependent protein phosphatase, is naturally myristoylated and can accept the 12-ADA tag without engineering [42, 43]. Similarly, protein kinase A (PKA) is a naturally myristoylated protein that carries out many functions in the cell including regulation of transcription factors [44], modulation of ion channel activity [45], control of receptor trafficking [45], and apoptosis [46]. Here, we demonstrate facile bioconjugation of azide-labeled CaM, CaN, and PKA due to their important role in intercellular communication as signaling proteins and enzymes [44] [47, 48]. Further, there are established methods for testing the activity of CaM, CaN [36] [49] [50], and PKA that we employ to ensure that our conjugation methods do not significantly impact protein function after conjugation to the nanoparticle surface.

Here, we demonstrate approaches to generate protein–particle conjugates that limit or reduce particle aggregation, provide site-specific conjugation of the protein to the surface, and maintain high levels of protein activity post-conjugation. We perform selective and N-terminal specific bio-orthogonal labeling of proteins of interest with 12-ADA during protein expression followed by *in situ* conjugation of N-terminal azide-labeled proteins onto gold nanoparticle (AuNP) surfaces and magnetic beads. CaM, CaN, and PKA are co-translationally labeled with 12-ADA in recombinant expression systems using the NMT-mediated chemoenzymatic tagging method [35, 36, 40]. Labeled CaM and CaN are then conjugated to the 100-nm AuNPs in different sample matrices, and PKA is conjugated to magnetic beads, using azide-alkyne cycloaddition reactions, a popular form of “click” chemistry [51, 52]. We demonstrate that azide-labeled proteins can be efficiently conjugated to AuNPs and magnetic beads directly from cell lysate, negating the need for a purification step and simplifying the generation of particle–protein conjugates. In addition, we demonstrate that click chemistry-functionalized AuNPs are highly stable and resist aggregation for at least 9 months. Furthermore, this conjugation method enables high levels of protein activity. Designing nanoparticle systems with 12-ADA or other site-specific azide-tagged proteins would provide new opportunities to generate hybrid materials for applications in biosensing, imaging, and nanomedicine.

## Material and methods

### Protein expression and labeling with human N-myristoyl transferase

We cultured BL21 (DE3)-competent *Escherichia coli* (New England BioLabs, Ipswich, MA) that had been co-transformed with two vectors: one for expression of human N-myristoyl transferase 1 (hNMT, pHV738 plasmid encoding human NMT1, and methionine aminopeptidase [53]) and a second vector for expression of (1) CaM that had been engineered to be a substrate of hNMT [35]; (2) human CaN (pET15b CanA CnB, a gift from Anjana Rao (Addgene plasmid #11787 [54])); (3) emerald green fluorescent protein (EmGFP) engineered to express the NMT recognition sequence from yeast ADP ribosylation factor (yARF-EmGFP) [36]; or (4) a the catalytic subunit of PKA (pRSETB PKA Cat was a gift from Susan Taylor (Addgene plasmid # 14920 [55]). Growth and expression in *E. coli* were performed in lysogeny broth (LB) supplemented with 100 μg mL^−1^ ampicillin and 50 μg mL^−1^ of kanamycin. Cultures were inoculated from glycerol stocks for overnight growth in 5 mL LB at 37°C and shaken continuously at 250 rpm (Forma Orbital Shaker, Thermo Electron Corporation, Waltham, MA). Cultures were then used to inoculate a 1 L culture and grown at 37°C until reaching an OD_600_ of 1 for CaM, CaN, and EmGFP and 0.8 for PKA. Protein expression was induced with 1 mM IPTG for CaM, CaN, and EmGFP and 0.4 mM for PKA. Simultaneously, either 12-ADA or myristic acid was added to a final concentration of 0.5mM, respectively (synthesized according to methods from Devadas et al. and Kulkarni et al. with slight modifications) [35] [56, 57]. For WT-CaM controls, a pET-15b vector with wildtype drosophila CaM (gift from Prof. Stephen Mayo [58, 59]) was used for co-transformation, and no 12-ADA was added at the induction step. Cells were harvested 3–4-h post-induction via centrifugation. Cell pellets were washed with cold 1X PBS, aliquoted, and stored at −80°C until use.

### Purification of CaM

Phenyl Sepharose 6 Fast Flow High Substitution (Product # GE17-0973-05, Sigma-Aldrich, St. Louis, MO) was used to purify N_3_-CaM and WT-CaM according to established protocol, taking advantage of the Ca^2+^-dependent structural changes of CaM that expose hydrophobic regions involved in many binding dynamics [35]. Briefly, cell pellets were resuspended in CaM lysis buffer (50 mM HEPES (pH 7.5), 100 mM KCl, 1 mM EGTA, 1 mM EDTA, 1 mM DTT, 0.1 mM PMSF, Roche Complete Inhibitor Cocktail, 0.5–1 mg mL^−1^ lysozyme) and lysed with sonication for 3 min in 0.5 s pulses. Clarified lysate was obtained after centrifugation (12,000 × g for 20 min, 4°C) and incubated with Phenyl Sepharose 6 Fast Flow for 30 minutes. Flow through was collected, then applied to another column of Phenyl Sepharose 6 Fast Flow (pre-equilibrated in 50 mM HEPES (pH 7.5), 3 mM CaCl_2_, 0.1 mM PMSF), and incubated for 30 min. The flow through was collected, followed by 3 washes using 2 column volumes of Wash Buffer (50 mM HEPES (pH 7.5), 1 mM CaCl_2_) and further 3 washes with 2 column volumes of Wash Buffer with 500 mM NaCl. CaM proteins were eluted with 50 mM HEPES (pH 7.5) and 1.5 mM EGTA. Protein concentrations of elution fractions were quantified using Pierce 660 Protein Assay, and purity was evaluated with SDS-PAGE (4-20% Mini-PROTEAN®TGX™ Precast Gels, Bio-Rad, Hercules, CA). Elution fractions containing the highest concentrations of pure 12-DA and WT-CaM were pooled and dialyzed in 20 mM HEPES (pH 7.5). The final protein concentration was brought to 1 mg mL^−1^ for conjugation. CaN was purified according to previously established protocols from Mondragon *et al.* [54].

### Functionalization of gold nanoparticles and magnetic beads

#### ***Functionalization of AuNP with DBCO***

**Fig. 1:**
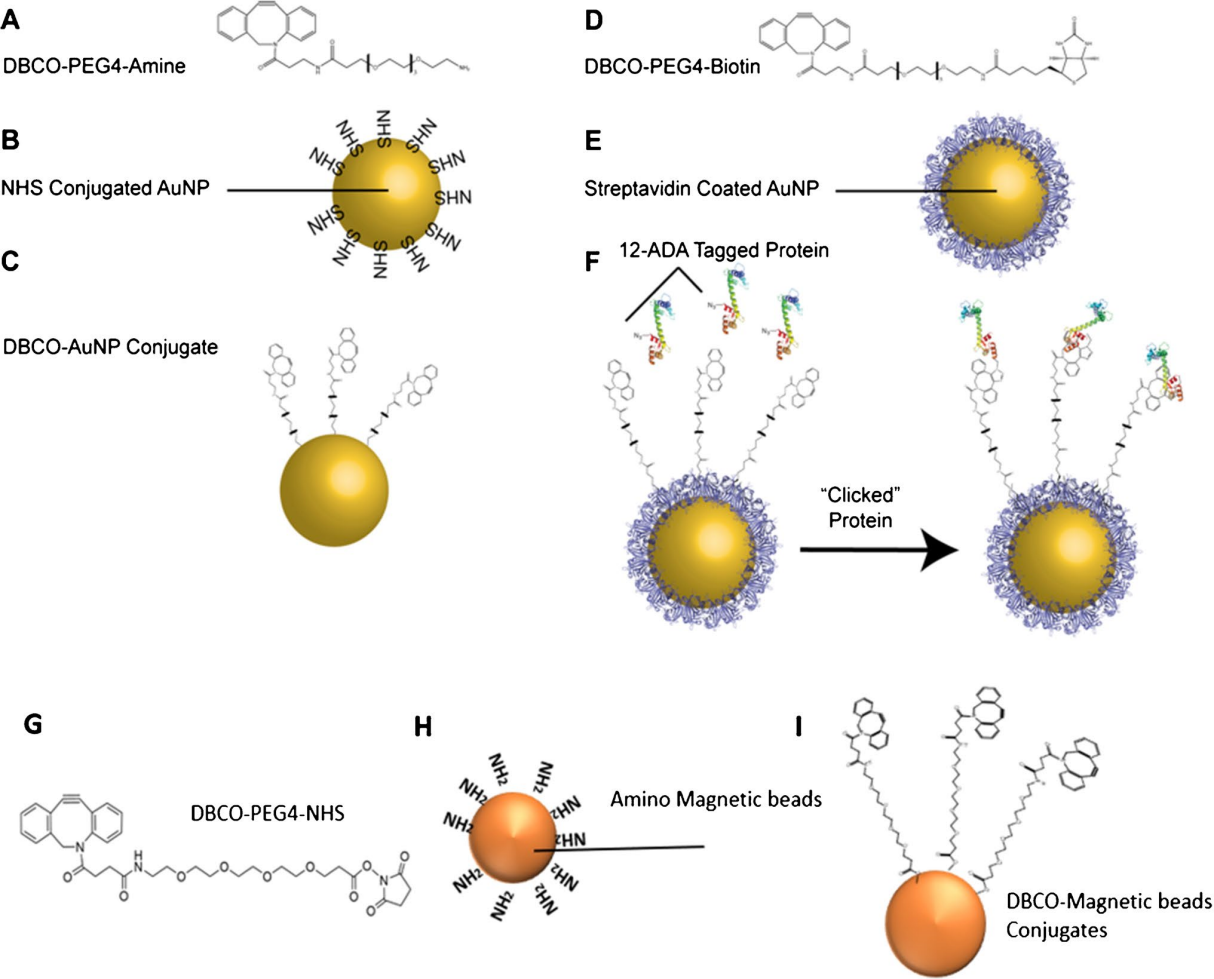
DBCO AuNP/magnetic bead functionalization. **A** DBCO-PEG4-amine and **B** NHS-conjugated AuNPs are combined to form a **C** DBCO-functionalized AuNP. **D** DBCO-PEG4-biotin are combined with **E** streptavidin-coated AuNPs to make stable conjugates. **G** DBCO-PEG4-NHS and **H** amino-functionalized magnetic beads are combined to form **I** DBCO-magnetic bead conjugates. **F** These DBCO-functionalized particles are then combined with 12-ADA-tagged recombinant proteins to produce protein–particle conjugates via strain-promoted click chemistry

A 100-μL aliquot (OD_572_ = 20, and ~7.68E+10 particles per mL of 100 nm NHS-activated AuNPs (Gold Nanoparticle Kit (10 reactions, Cytodiagnostics, Ontario, Canada, Fig. 1B)) were incubated with 100 μL 1.25 mM DBCO-PEG4-amine (Click Chemistry Tools, Scottsdale, AZ in Fig. 1A) dissolved in 50% v/v DMSO and 20 mM HEPES (final concentration of HEPES 10mM) for 2 h at room temperature with gentle rotation. Following incubation, free NHS groups were inactivated through the addition of 10 μL quenching buffer (provided by the manufacturer) according to Cytodiagnostic’s protocols, forming DBCO terminated 100 nm AuNPs (Fig. 1C). The reaction was centrifuged for 20 min at room temperature at 1000 × g and resuspended in 20 mM Tris pH 7.5, 150 mM NaCl at a final OD_572_ of 1.5. Particles were immediately measured for size and polydispersity index (PdI) with a Malvern Zetasizer Nano ZS90 (Malvern, UK). Alternatively, 40 μL of 100-nm streptavidin-conjugated gold nanoparticles (Fig. 1E) at a concentration of OD_572_ = 3 (Cytodiagnostics, Ontario, Canada) were incubated with rotation with 2 μL of 1.25 mM DBCO-PEG4-biotin (Click Chemistry Tools, solubilized in 100% DMSO, Fig. 1D) for 2 h at room temperature. The particles were then diluted by adding a 1:1 solution volume of buffer (20 mM Tris pH 7.5, 150 mM NaCl).

#### *Functionalization of AuNP with alkyne*

Forty-eight microliter of 0.5 M alkyne-PEG4-amine (Fig. 3A) dissolved in DMSO and 60 μL of 20 mM HEPES pH 7.4 were combined. Ninety microliter of this mixture was added to a lyophilized aliquot of 100 nm NHS-activated AuNPs (Gold Nanoparticle Kit (10 reactions), Cytodiagnostics, Ontario, Canada, Fig. 3B) for final AuNP concentration of OD_572_ = 22. The particles were incubated for 4 h at room temperature with gentle rotation. Termination of the reaction was achieved through the addition of 10 μL quenching buffer (as provided by the manufacturer) according to Cytodiagnostic’s protocols, forming alkyne terminated 100 nm AuNP (Fig. 3C) with a final volume 100 μL, and OD_572_ = 20. Following this, the particles were centrifuged for 20 min at 2000 × g, and the supernatant was replaced with 10 μL of 2 mg/mL BSA solution diluted in 20 mM HEPES. Particles were incubated at room temperature for 30 min to block any exposed particle surfaces. The particles were centrifuged again for 20 min at 2000 × g, and the supernatant removed. Particles were stored at 4°C.

#### *Functionalization of magnetic beads*

Six hundred microliter of Spherotech Amino Magnetic beads (#AMS-40-10, Spherotech supplied as 2.77E8 beads/mL, Fig. 1G) was equilibrated into 20-mM HEPES buffer and washed three times and brought to a final volume of 600 μL. Beads were functionalized with 9 μL of 100 mM DBCO-PEG4-NHS ester (NHS-DBCO, #A134-2, Click Chemistry Tools, Fig. 1H) for a final concentration of 2 mM DBCO and rotated end over end at room temperature for 30 min. Beads were washed with 600 μL TBS (50mM Tris (pH 7.5), 150mM NaCl) four times and brought to a total volume of 600 μL following washes. Six hundred microliter of 2 mg/mL BSA in TBS was added to beads and rotated end over end at 4°C for 30 min and washed two times with TBS and brought to a total volume of 600 μL. These functionalized beads (Fig. 1I) were used for conjugation and enrichment of 12-ADA-labeled protein.

### Conjugation of proteins to functionalized nanoparticles and magnetic beads

#### ***Conjugation of functionalized AuNP***

For conjugating the protein to the DBCO-functionalized AuNPs, strain-promoted alkyne-azide cycloaddition (SPAAC) click chemistry was employed. The 42 μL of DBCO-functionalized AuNP particles was diluted to a final volume of 80 μL in 20mM Tris pH 7.5, 150 mM NaCl. Following this, 10 μL of purified N_3_-CaM or WT-CaM, N_3_-CaN, and N_3_-EmGFP (initial protein concentration of 0.1 mg mL^−1^) was added to the particles, separately and incubated for 2 h on a rotator at room temperature (a schematic of this procedure is presented in Fig. 1F). Measurement of the size of the particles before and after was determined by dynamic light scattering (DLS) on the Malvern Zetasizer ZS90.

#### ***Conjugation of functionalized magnetic beads***

Cells containing overexpressed 12-ADA PKA or Myr PKA or cells with no overexpressed proteins were lysed. One hundred thirty-three microliter of 1.0 mg/mL lysates containing either 12-ADA PKA, Myr PKA, or no overexpressed protein was added to the 133μL of cold (4°C), freshly prepared DBCO-functionalized beads, and allowed to react overnight at 4°C. Beads were then washed to remove contaminant proteins: twice with 1 mL 1 × TBST, twice with 1 mL 50 mM Tris; 0.5 M NaCl (4°C), and then three times with 1 mL TBS (4°C). Washes were conducted at 4°C to maintain protein function.

To verify that only 12-ADA-tagged proteins were bound to the particle surface, a series of controls were used for comparison. The 12-ADA-tagged proteins used in the experiments were N_3_-CaM, N_3_-CaN, and N_3_-EmGFP. Control groups were purified WT-CaM (purchased from Enzo Scientific) and myristoylated CaN (Myr-CaN). The 12-ADA-tagged proteins (either purified or overexpressed in cell lysates) or the controls (WT-CaM or Myr-CaN) were added to the alkyne-functionalized AuNPs in the presence of reagents for copper(I)-catalyzed azide-alkyne cycloaddition (CuAAC): 30 μL of protein (between 0.1 and 1 mg/mL concentration), 20 μL of 0.5 M iodoacetamide, 40 μL of 100 mM amino guanidine, 10 μL of 400 mM sodium ascorbate, 40 μL of 50 mM THPTA, and 16 μL of 25 mM CuSO_4_. The AuNPs were incubated with the 12-ADA protein solution overnight at 4°C. After overnight incubation, the nanoparticles were washed with 20 mM HEPES via centrifugation for 20 min at 2000 × g and stored again in 20 mM HEPES (pH 7.4) at 4°C until used for measurements.

### Dynamic light scattering

DLS measurements of particle size and polydispersity (PdI) of DBCO- and alkyne-functionalized AuNPS at an OD_572_ of 1.5 were performed with the Malvern Zetasizer Nano ZS90 according to manufacturer’s instructions. One milliliter disposable polystyrene cuvettes were used (DTS0012, Malvern Instruments, Westborough, MA) for measurements.

### Mass spectrometry

Mass spectrometry was performed to validate the presence of N_3_-CaM and N_3_-CaN bound to the nanoparticle surface. Three groups were analyzed with mass spectrometry; N_3_-CaM-conjugated AuNPs, N_3_-CaN-conjugated AuNPs, and a control of alkyne-functionalized AuNPs blocked with 1 mg/mL BSA. All three groups were subjected to trypsin digestion (Pierce™ MS-grade trypsin protease, ThermoFisher, Erie, NY). One microgram of trypsin was added to each AuNP solution (100 μL at OD_572_ = 20) and incubated at 37°C overnight. Samples were centrifuged (1000 × g, 5 min), and the supernatant was removed for processing and mass spectrometry analysis at the Bindley Bioscience Center at Purdue University’s Discovery Park.

### UV-Vis spectroscopy

Particle absorbance was measured with UV-Vis spectroscopy with a Nanodrop 2000 (Thermo Scientific, Erie, NY). Briefly, the spectrometer was blanked with 20 mM HEPES pH 7.4 buffer, and absorbance scans from 400 to 750 nm were performed with particles suspended in 20 mM HEPES pH 7.4 (at an approximate concentration of OD OD_572_ = 20). Maximum peak absorbance for each sample was determined to investigate red or blue shifts after particle conjugation occurred.

### Agarose gel electrophoresis

A 0.5% w/v agarose gel in 1X TAE was used for investigating size separation of conjugated nanoparticles. All particles underwent a buffer exchange in Elution Buffer (QIAGEN, Hilden, Germany). Particle samples were combined with Gel Loading Dye, Purple (6X) at a ratio of 13 μL sample to 2 μL dye (New England Biolabs, Ipswich, MA). The gel was run at 150V for 40 min and imaged on the Azure c400 (Azure Biosystems, Inc., Dublin, CA) with visible and fluorescent light (Cy2) to investigate fluorescence signal for the emGFP samples (excitation/emission, 487/509 nm).

### Protein activity assay

#### ***Enzymatic activity of N***_***3***_***-CaM-functionalized AuNP***

An Enzo calcineurin phosphatase protein activity assay (cat# BML-AK804, Farmingdale, NY) was used to probe the ability of N_3_-CaM to activate CaN phosphatase activity after conjugation to the 100 nm AuNPs. The protein activity assay was followed according to manufacturer’s instructions. In brief, 33 μL at an OD_572_ = 20 of N_3_-CaM-conjugated particles was combined with assay buffer contents (25 μL 1X assay buffer with 0.5 μM diluted calmodulin, 5 μL CaN at 40U per reaction, 10 μL of N_3_-CaM-conjugated AuNPs at OD20, and 10 μL 0.75 mM phosphopeptide substrate) for 10 min followed by centrifugation at 16,000 × g for 5 min. Supernatants were added to a 96-well half-volume assay plate along with serial dilutions of PO_4_^3−^ in assay buffer (0–2 nM) for the standard curve. One hundred microliter of BIOMOL Green reagent was added to all sample and standard wells and incubated for 30 min. Absorbance at OD_620_ was measured, and linear interpolation against the standard curve was used determine the amount of PO_4_^3−^ released in the phosphatase assay.

#### ***Enzymatic activity of PKA-functionalized beads***

An ELISA-based PKA kinase activity kit (#ADI-EKS-390A, Enzo) was used to measure activity of PKA-functionalized beads. Thirty microliter of DBCO-magnetic beads that were conjugated to protein in the presence of clarified cell lysate containing either recombinant 12-ADA PKA, Myr PKA, or no overexpressed recombinant protein (E coli lysate) was added to each PKA-specific substrate coated well along with 10 μL of 1 mg/mL ATP and incubated for 90 min at 30°C. To obtain activity of PKA-functionalized beads as a function of ATP concentration, 30 μL of 12-ADA PKA beads was added to wells along with 10 μL of ATP at different concentrations and incubated for 90 min at 30°C. For the inhibitor dose–response assay, 30 μL of 12-ADA PKA beads were mixed using a thermomixer with 5 μL of PKA fragment inhibitor (P6062, Sigma-Aldrich), at different concentrations, for 10 min prior to addition to the wells. The beads were then added to each coated well with 5 μL of 2 mg/mL ATP and incubated at room temperature for 10 min. Antibody detection of phosphorylated substrate was performed according to manufacturer’s instructions. Absorbance was measured at 450 nm. GraphPad Prism (La Jolla, CA) software was used for data analysis and generating graphs. Data were fit to a sigmoidal curve to obtain EC50 and IC50 values (Eqn 1 and 2, respectively):

1 $$Y=\frac{100}{1+{10}^{\left( LogEC50-X\right)}}$$

2 $$Y=\frac{100}{1+{10}^{\left(X- LogIC50\right)}}$$

## Results and discussion

### Functionalization of AuNPs/magnetic beads with DBCO

**Fig. 2:**
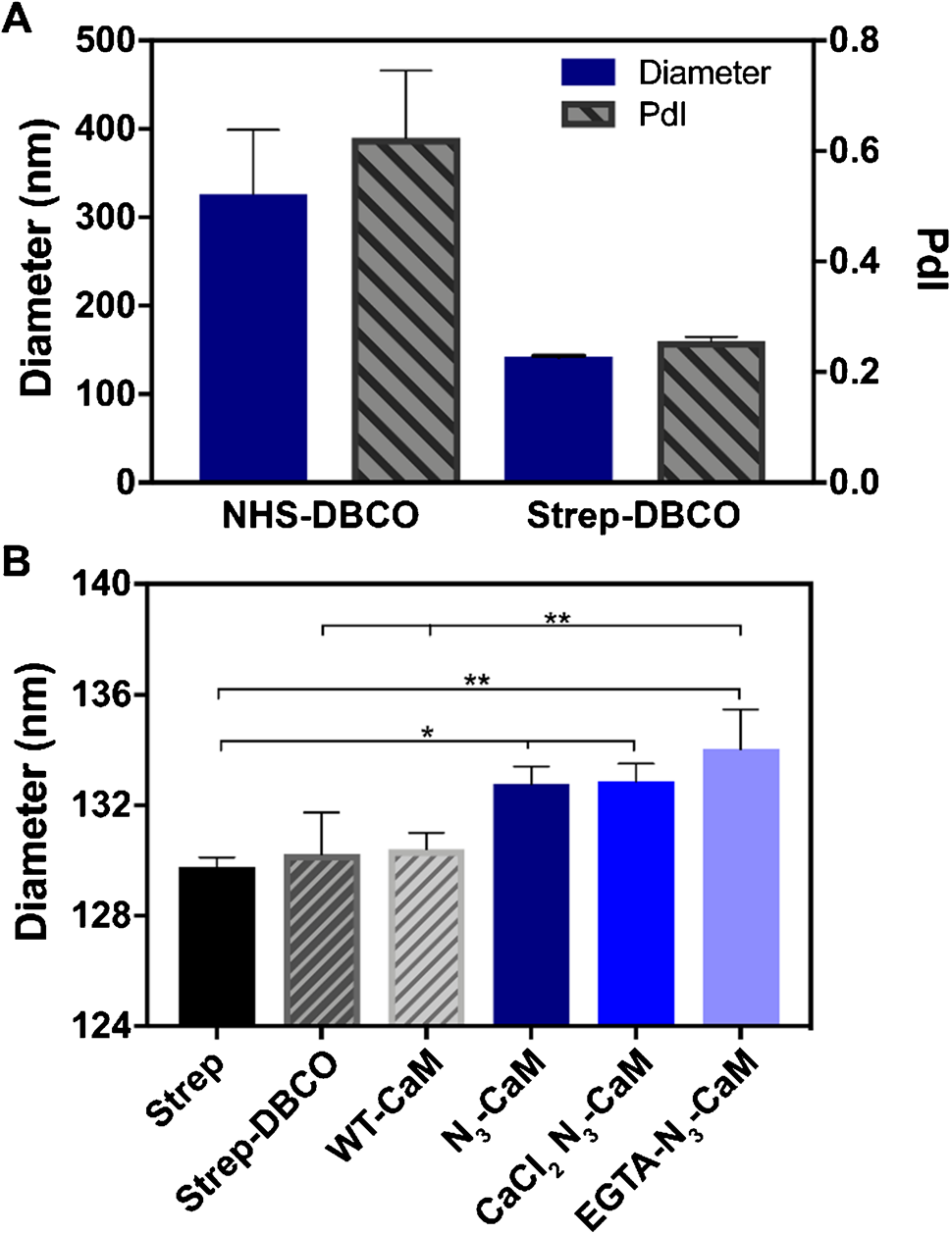
DBCOAuNP sizing and polydispersity (PdI). **A** Size of DBCO-functionalized AuNPs measured with dynamic light scatter (DLS). Primary amine conjugation of 100 nm NHS AuNPs with amine-PEG4-DBCO (NHS-DBCO) diameter = 300 nm ± 72 nm (left ordinate axis) and PdI = 0.6 ± 0.12 (right ordinate axis), *n* = 6. One hundred nanometer streptavidin AuNPs after conjugation to biotin-PEG4-DBCO (Strep-DBCO); diameter = 130 nm ± 1.5nm and PdI = 0.2 ± 0.01, *n* = 6. **B** Size of AuNPs after protein functionalization. One hundred nanometer streptavidin AuNPs (Strep); streptavidin AuNPs conjugated to biotin-PEG4-DBCO (Strep-DBCO); Strep-DBCO particles that underwent click chemistry conjugation (click) in the presence of wildtype calmodulin (CaM) (WT-CaM); Strep-DBCO particles that underwent click in the presence of azide (N_3_)-functionalized calmodulin (N_3_-CaM); Strep-DBCO particles that underwent click in the presence of CaCl_2_ and N_3_-CaM (CaCl_2_ N_3_-CaM); and Strep-DBCO particles that underwent click in the presence of EGTA and N_3_-CaM (EGTA N_3_-CaM). (*n* = 3, ** *p* < 0.01, * *p* < 0.05). Error bars are standard deviation

Strain-promoted alkyne-azide cycloaddition (SPAAC) is the generally preferred method of click conjugation when maintenance of protein enzymatic functionality is desired. This is due to the reaction being copper-free; along with its known cytotoxicity, excess copper may adversely affect metal co-factors that are key to may enzymatic functions [60, 61]. Therefore, we functionalized AuNPs with dibenzocyclooctyne (DBCO) (Fig. 1A–C) by incubating amine-PEG4-DBCO (Fig. 1A) with 100 nm N-hydroxysuccinimide (NHS)-functionalized AuNPs (Fig. 1B), taking advantage of the primary amine chemistry between the NHS of the particle and amine linked to the DBCO molecule (Fig. 1C). As an alternative approach to functionalizing AuNPs with primary amine chemistry, we conjugated DBCO to AuNP via streptavidin–biotin affinity interaction (Fig. 1D–E). A heterobifunctional PEG terminated with biotin and DBCO (biotin-PEG4-DBCO, Fig. 1D) was incubated with 100 nm streptavidin-coated AuNPs (Fig. 1E) and 0.1% w/v BSA as a stabilizing reagent to inhibit irreversible particle aggregation. The AuNP samples were, on average, smaller in particle diameter (on the order of 130 nm) and had a lower particle dispersity index (PdI) (on the order of 0.2) with the biotin-PEG4-DBCO conjugation as compared to the primary amine conjugation (Fig. 2A). One hundred nanometer NHS AuNPs with amine-PEG4-DBCO (NHS-DBCO) resulted in high levels of aggregation as indicated by an increase in average diameter of almost threefold (diameter = 300nm ± 72nm, PdI = 0.6 ± 0.12). The 100-nm streptavidin AuNPs after conjugation to biotin-PEG4-DBCO (Strep-DBCO) had an average diameter of 130 nm ± 1.5nm and PdI = 0.2 ± 0.01. Collectively, these findings suggest that DBCO-conjugated, streptavidin-coated AuNPs stabilized with BSA do not aggregate and are suitable for downstream SPAAC click chemistry bioconjugation.

Similar to the functionalization of AuNP with DBCO, amino magnetic beads (Fig. 1G) were functionalized with DBCO-PEG4-NHS ester (NHS-DBCO, #A134-2, Click Chemistry Tools (Fig. 1G–H). The size and polydispersity of the AuNP post-DBCO conjugation were measured via dynamic light scattering (DLS). Particle diameter was larger than expected (>300 nm), and samples had a high polydispersity index (PdI > 0.6) (Fig. 2A). This result indicates a significant amount of aggregation of the DBCO AuNPs.

### Conjugation of DBCO-functionalized AuNPs with azide-tagged protein

Having stably functionalized 100 nm AuNPs with DBCO, we sought to conjugate purified azide-tagged CaM (N_3_-CaM, Figure S1) to the AuNPs. Successful conjugation of the N_3_-CaM to the DBCO on the nanoparticles is reflected by an increase in their size as measured with dynamic light scattering (DLS) (Fig. 2B). We measured six different experimental groups to gain insight into the successful conjugation of the N_3_-CaM to the AuNPs and the influence of protein–nanoparticle conjugation on N_3_-CaM conformation. Of the six experimental groups (Fig. 2B), three control groups consisted of (1) pure 100-nm streptavidin-conjugated nanoparticles (Strep, labeled black in Fig. 2B); (2) 100-nm streptavidin particles functionalized with DBCO and stabilized with BSA as described above (Strep-DBCO, dark gray, Fig. 2B); and (3) streptavidin particles treated with DBCO and incubated with purified wildtype CaM (WT-CaM, light gray, Fig. 2B). This last control was introduced to access whether non-specific binding of CaM occurs (Fig. 2B). Within the three sample groups, we investigated the effect of calcium (Ca^2+^) binding to the conjugated N_3_-CaM, since CaM conformation changes between the Ca^2+^ unbound (apo) and Ca^2+^ bound states [62, 63]. These states reflect a measurable change in its hydrodynamic radius (the Stokes radius of CaM is 2.48 ±0.9 nm in apo state and 2.45±0.4 nm in its Ca^2+^-loaded state). Purified N_3_-CaM was conjugated to DBCO-treated streptavidin particles under varying buffer conditions: (1) in 20 mM HEPES buffer (N_3_-CaM Fig. 2B, dark blue); (2) in the presence of CaCl_2_ (CaCl_2_ N_3_-CaM Fig. 2B, blue); and (3) in the presence of EGTA (EGTA-N_3_-CaM Fig. 2B, light blue) to chelate Ca^2+^ and leave CaM in its apo state.

As anticipated, the streptavidin-coated particles (labeled Strep) had the smallest diameter of the AuNP different groups (Fig. 2B). The addition of DBCO to the streptavidin-coated particle surface (labeled Strep-DBCO) resulted in a slightly larger average particle diameter, although not statistically significant. Addition of WT-CaM to the particles showed no statistically significant difference when compared to unconjugated streptavidin-coated particle (Strep) or labeled Strep-DBCO particles, suggesting lack of non-specific binding. In contrast, the addition of N_3_-CaM to the DBCO-conjugated particles (N_3_-CaM) resulted in a discernable and statistically significant change in particle diameter (compared to the streptavidin control group * *p* < 0.05).

Next, we studied the effect of Ca^2+^ on the conformation of CaM. Excess CaCl_2_ in the N_3_-CaM solution (labeled CaCl_2_-N_3_-CaM in Fig. 2B) resulted in a particle diameter that was similar to the N_3_-CaM group. However, chelating Ca^2+^ with EGTA (labeled EGTA-N_3_-CaM, Fig. 2B) produced a statistically significant increase in hydrodynamic radius. Taken together, these results indicate the possibility that under our purification and conjugation conditions, N_3_-CaM is bound to Ca^2+^ or in a similar conformational state when conjugated to the AuNPs, which is important for understanding the behavior and size of CaM AuNPs in binding events downstream.

### Conjugation of alkyne-functionalized AuNPs with azide-tagged protein

**Fig. 3:**
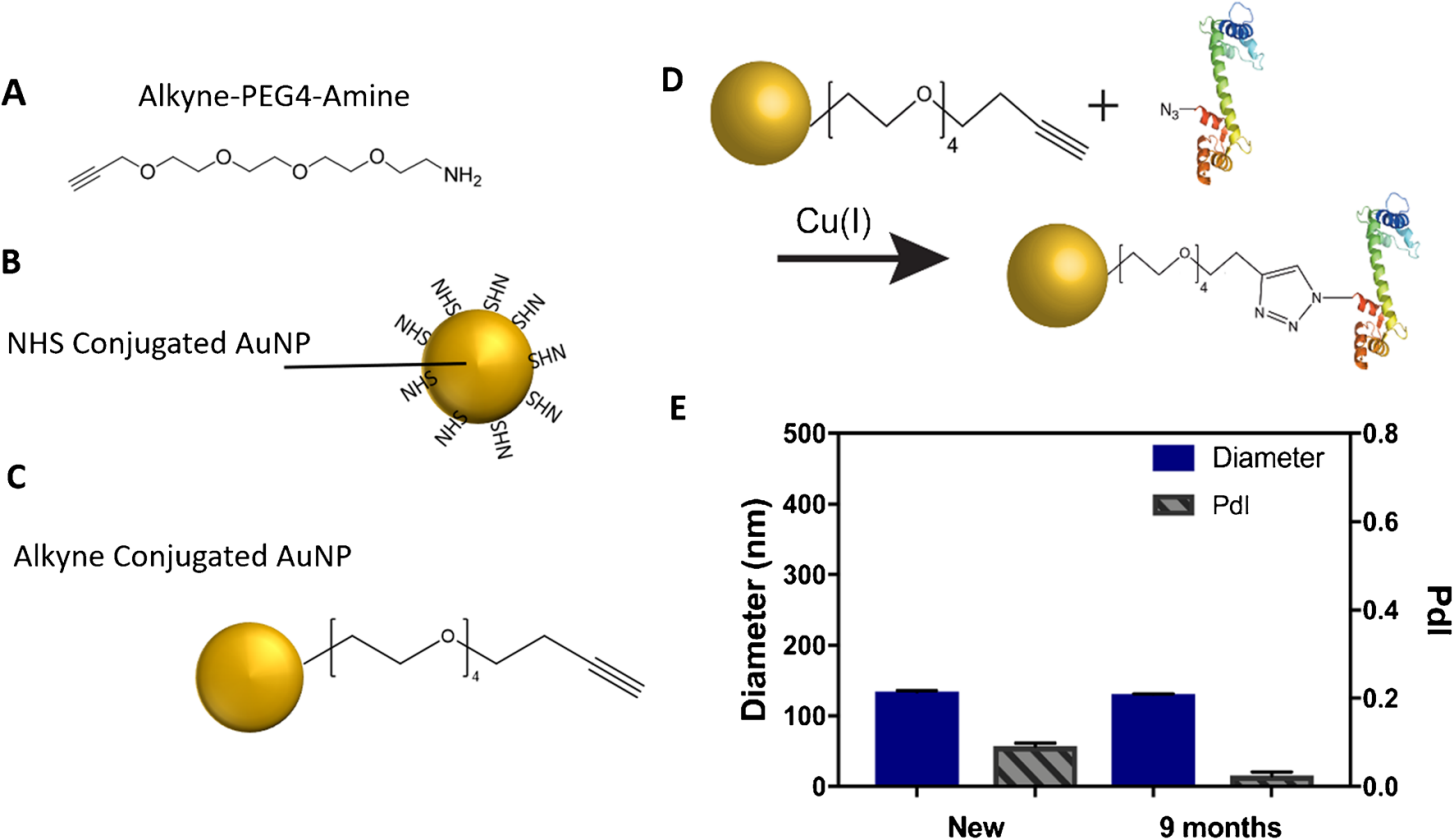
Alkyne-conjugated AuNPs. **A** Alkyne-PEG4-amine and **B** NHS-functionalized AuNPs were reacted to form **C** alkyne-functionalized AuNP. **D** Alkyne-functionalized AuNPs are conjugated to azide (N_3_)-labeled proteins in the presence of copper (Cu(I)) to covalently bind N_3_-tagged protein to the AuNPs. **E** Dynamic light scatter (DLS) measurement of particle diameter. There was not statistically significant difference in the average particle diameter of alkyne-conjugated AuNPs that were made fresh (new, diameter = 134 ± 1.9 nm (left ordinate axis) and PdI = 0.1 ± 0.006 (right ordinate axis)) compared to those that were stored for 9 months (9 months; diameter = 131.2±0.4 (left ordinate axis) and PdI = 0.025±0.008 (right ordinate axis)) (*n* = 3)

Although CaM was successfully conjugated via the streptavidin–biotin interaction and SPAAC, this approach was not deemed feasible for protein conjugation with complex sample matrices, such as cell lysates, where endogenous biotin and streptavidin may be present. CuAAC reaction is fast and highly selective, and in the absence of copper, this reaction become very slow and non-selective [64]. Therefore, we conducted subsequent experiments using copper-catalyzed azide-alkyne click chemistry (CuAAC). Alkyne-PEG4-amine (Fig. 3A) was reacted with NHS-functionalized AuNPs (Fig. 3B–C) to prepare them for conjugation with azide-functionalized proteins (Fig. 3D). After conjugation of the alkyne on the particle surface, the average size of the AuNPs was approximately 134 ± 1.9 nm with a PdI of 0.1 ± 0.006 (Fig. 3E). Additionally, we investigated the stability of these same alkyne-conjugated AuNPs after storage at 4°C for 9 months. The particles remained stable, showing low levels of aggregation with a PdI of 0.025 ± 0.008 (Fig. 3E). It should also be noted that stabilizing agents, such as BSA, were not contained in the solution and are therefore not necessary for maintaining particle stability. Indeed, our DLS data suggested that alkyne-conjugated AuNPs are more stable than the DBCO AuNPs. These results indicate promise in using CuAAC chemistry to covalently conjugate 12-ADA-tagged proteins onto the nanoparticle surfaces.

We investigated the feasibility of conjugating 12-ADA-tagged proteins onto AuNPs directly from *E. coli* lysates. Employing this method negates the extra protein purification steps that are often needed in nanoparticle conjugation procedures. The schematic of the conjugation process is demonstrated in Fig. 3D; here, particles come in direct contact with clarified lysate, but only 12-ADA-tagged proteins (labeled with N_3_ in Fig. 3D) covalently bind to the particles, and the rest of the clarified lysate can be washed away.

We have previously engineered emerald green fluorescent protein (emGFP) to carry an N-terminal 12-azidododecanoic acid (12-ADA) tag (N_3_-GFP) [36]. To confirm that N_3_-GFP was the only protein in lysate that can undergo CuAAC, *E. coli* lysate containing overexpressed and tagged N_3_-GFP was subjected to a fluorophore click reaction, followed by SDS-PAGE analysis (Fig S3).

**Fig. 4:**
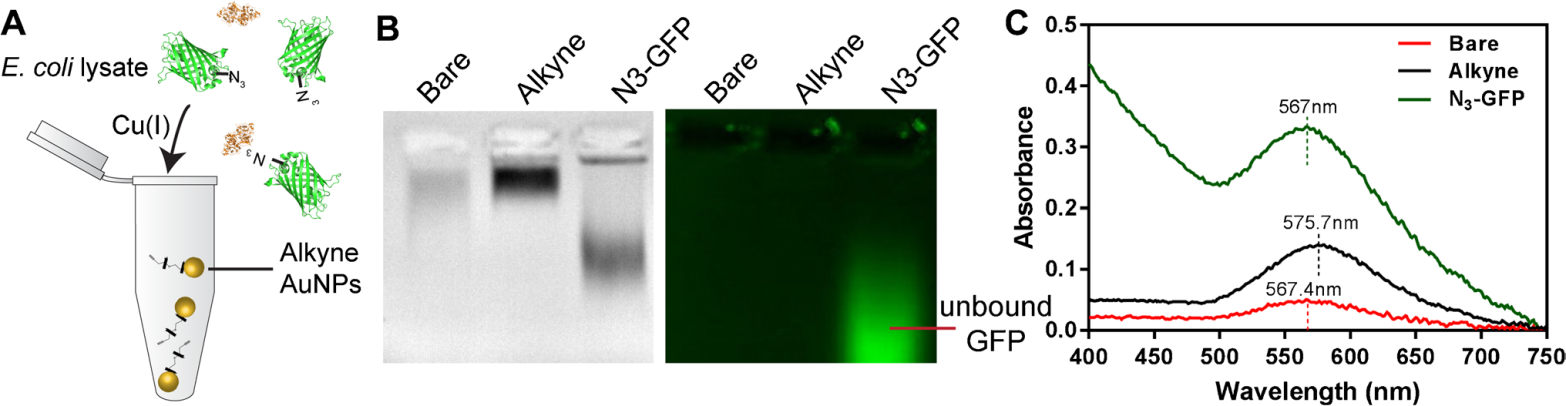
Conjugation of N_3_-GFP to AuNPs directly from cell lysates. **A** Alkyne-functionalized AuNPs undergo CuAAC in the presence of *E. coli* lysate with N_3_-tagged engineered proteins (in this schematic, N_3_-GFP, green, is pictured). **B** One hundred nanometer AuNPs with different treatments were run through agarose gel electrophoresis. AuNPs with clicked with N_3_-GFP show further migration down the gel than the alkyne and bare nanoparticles (left gel image). When the same gel is imaged for fluorescence, the unbound N_3_-GFP (green) is visible as a green band propagated further down the gel (right gel image). Note that the fluorescence of conjugated N_3_-GFP is quenched by the AuNPs. **C** Representative traces of UV-Vis spectroscopy of bare (red), alkyne-conjugated (black), and N_3_-GFP-conjugated AuNPs (green). There is a red shift in the peak of the absorbance spectra after the addition of the alkyne. Then there is a blue shift of the spectra after the addition of the N_3_-GFP to the particle surface

We investigated the conjugation of N_3_-GFP to the 100 nm AuNPs by performing gel electrophoresis. A 0.5% w/v agarose gel was used to maintain a large enough pore size for electrophoretic mobility of the AuNPs down the gel lane. We used two control groups for agarose gel electrophoresis: bare AuNPs (Bare) and alkyne-conjugated AuNPs (Alkyne) and compared them to the N_3_-GFP-conjugated particles. The N_3_-GFP-conjugated particles propagated further down the gel lane (toward the positive electrode) than the respective control groups (Fig. 4B). The isoelectric point (pI) of emGFP is approximately 5.58 (calculated from the emGFP sequence using ProtParam tool on Expasy server) [65]; emGFP would add significant negative charge when conjugated to the AuNPs. The distinct banding pattern of the N_3_-GFP sample implies that the particles are more electronegative than the bare and alkyne control groups.

We then imaged the same agarose gel for fluorescence (Fig. 4B). Unconjugated (free) N_3_-GFP is seen only in the gel lane containing the N_3_-GFP AuNPs as a broad band that migrated further than the lane with N_3_-GFP AuNPs. Note that in the fluorescent image, there is no green fluorescence where the N_3_-GFP AuNP band is visible in the brightfield gel image. This is to be expected considering the Förster resonance energy transfer (FRET) quenching effect that occurs when the emGFP interacts with metallic nanoparticles [66].

Further, to analyze each step of N_3_-GFP conjugation onto alkyne-functionalized 100 nm AuNPs, UV-Vis spectroscopy was used to measure the absorption spectra of two control groups: bare AuNPs (bare) and alkyne-conjugated AuNPs (Alkyne), to that of N_3_-GFP conjugated to AuNPs in clarified *E. coli* lysate containing over expressed N_3_-GFP (Fig. 4C). There was a red shift (higher wavelength shift) in the peak absorbance of the UV-Vis spectra of the alkyne-conjugated AuNPs relative to the bare AuNPs. This shift indicated the successful addition of the amine-PEG4-alkyne onto the AuNPs. When the N_3_-GFP was conjugated onto the AuNP surface, there was an opposing blue shift (lower wavelength shift) relative to the alkyne group alone. This indicated that the N_3_-GFP was successfully conjugated on the particle surface. Additionally, due to the absorbance properties of GFP, the UV-Vis data displayed a high absorbance signal between the 400 and 500 nm wavelength (Fig. 4C).

**Fig. 5:**
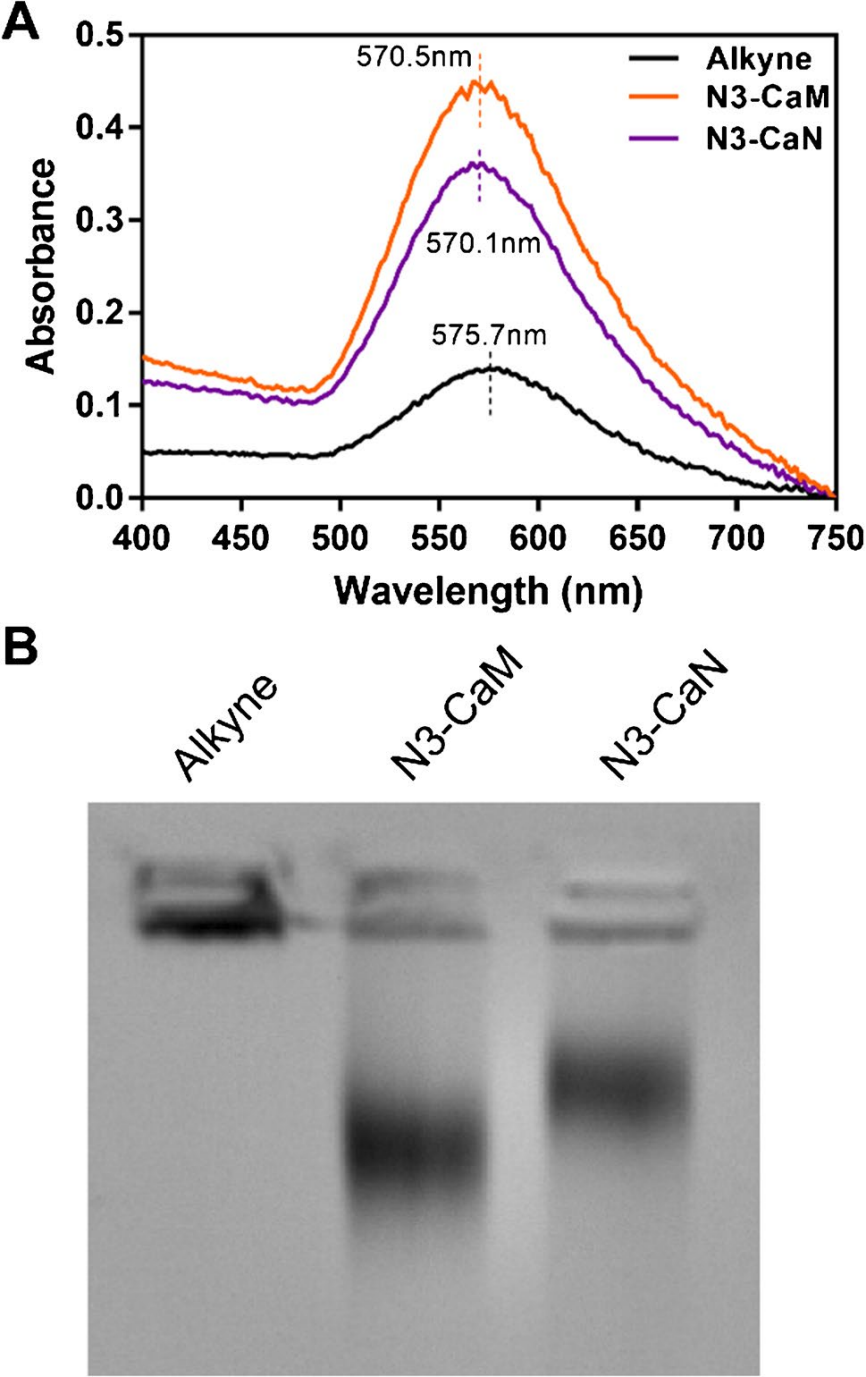
Conjugation of N_3_-CaN and N_3_-CaM to AuNPs directly from cell lysates. **A** N_3_-CaM- and N_3_-CaN-conjugated particles show blue shifts in the UV-Vis spectra as compared to alkyne particles. **B** The conjugation of N_3_-CaM and N_3_-CaN from *E. coli* lysate is further confirmed with agarose gel electrophoresis, showing distinctly different banding on the gel compared to each other and the alkyne-coated AuNP

After performing proof-of-principle measurements with the N_3_-GFP particles, we used the same approach of agarose gel electrophoresis and UV-Vis spectroscopy to verify CuAAC-mediated conjugation of N_3_-CaM and N_3_-CaN from *E. coli* lysate on AuNPs. AuNPs were treated to CuAAC in the presence of *E. coli* lysate with overexpressed and tagged N_3_-CaM and N_3_-CaN(S2). UV-Vis wavelength scans show that there is a blue spectral shift of the N_3_-CaM and N_3_-CaN-conjugated AuNPs relative to the control alkyne-functionalized AuNPs (alkyne) (Fig. 5A). This spectral shift is like what was observed with the N_3_-GFP results (Fig. 4C).

Agarose gel electrophoresis (Fig. 5B) analysis showed that alkyne-conjugated particles (alkyne) show the least amount of electrophoretic mobility relative to the sample groups. N_3_-CaM-conjugated particles propagate the furthest down the gel lane toward the positive electrode. The N_3_-CaN particles also migrate down the gel lane; however, there is a clear difference in the location of the particle band as compared to the N_3_-CaM band (MW of CaM = 16 kDa versus MW of the CaN heterodimer = 80 kDa). Taken together, these results show that recombinantly expressed and 12-ADA-tagged proteins can be covalently conjugated to AuNPs via CuAAC directly from *E. coli* lysates without the need for prior protein purification.

### Copper-catalyzed conjugation methods

In addition to the SPAAC chemistry, we investigated copper(I)-catalyzed alkyne-azide cycloaddition (CuAAC) click chemistry of the 12-ADA-tagged proteins onto 100-nm AuNP surfaces. Although SPAAC methods are sometimes preferred due to the copper-free reaction conditions, CuAAC methods can be favorable because they employ smaller linking molecules in reactions (alkyne versus DBCO). DBCO molecules are large and hydrophobic and, as seen in Fig. 1A, can cause the nanoparticles to aggregate. To avoid this, we implemented CuAAC in subsequent experiments.

To examine the efficiency of CuAAC conjugation for different proteins, we “clicked” N_3_-CaM or azide-labeled calcineurin (N_3_-CaN, Figure S1) proteins onto the AuNP surfaces with CuAAC. To determine the specific conjugation of N_3_-CaM and N_3_-CaN onto the particle surface, mass spectrometry (MS) was performed. We analyzed three groups against the human proteome library: N_3_-CaM, N_3_-CaN, and a control group containing a 1% BSA solution (but no 12-ADA-tagged protein). The MS results, presented in Table 1, show that there are a significant number of unique N_3_-CaM and N_3_-CaN peptides that are present in the nanoparticle samples. These unique peptides do not appear in the BSA control group. Further, the percentage of peptide intensity unique to CaM and CaN was 94% and 66%, respectively, affirming that the target proteins were most prevalent in these samples. Therefore, we determine that both N_3_-conjugated proteins successfully click onto the AuNP surface through CuAAC.

**Table 1:** Mass spectrometry results of N_3_-CaM (CaM) and N_3_-CaN (CaN) bound to clicked AuNPs as compared to the control group of non-specifically adsorbed protein (BSA)

	N_3_-CaM	N_3_-CaN	BSA
Total peptide # (MS/MS)	122	157	15
N_3_-CaM peptide # (MS/MS)	18	0	0
N_3_-CaN peptide # (MS/MS)	0	72	0

**Fig. 6:**
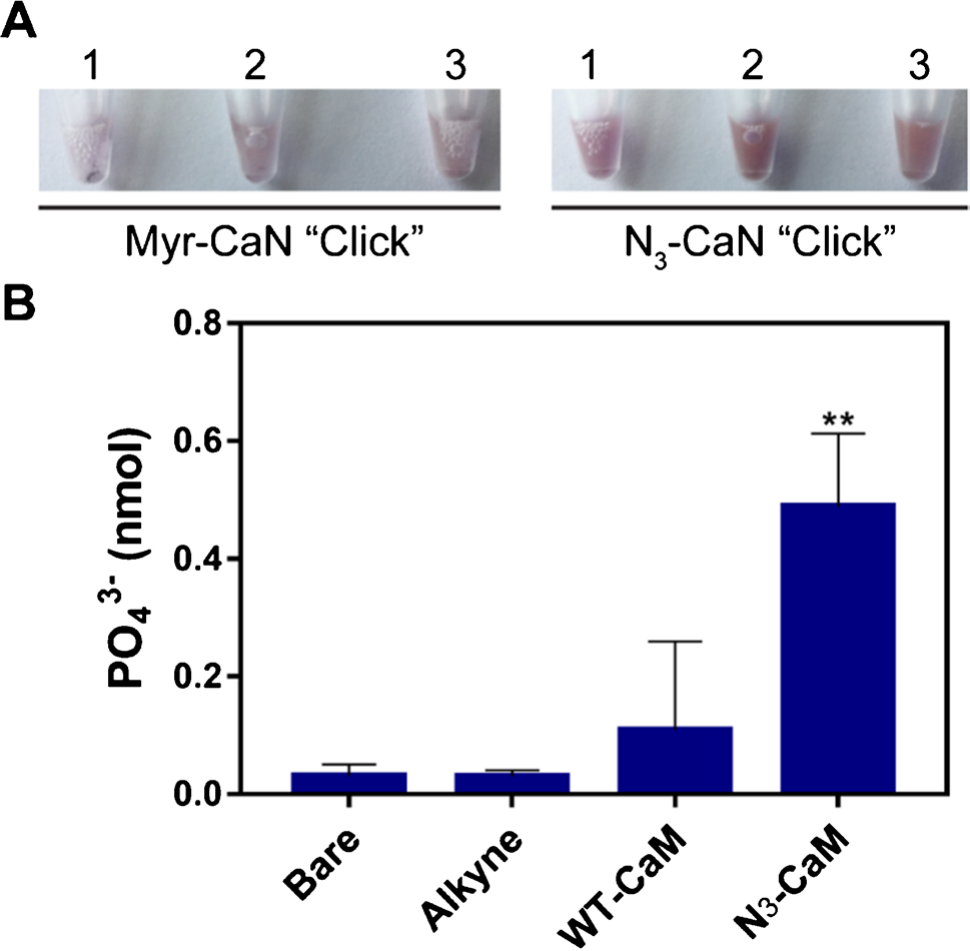
AuNP stability and protein activity from CuAAC. **A** Proteins and nanoparticles that undergo CuAAC procedures show aggregation when the protein is not N_3_ tagged versus when the protein is N_3_-functionalized. For example, with myristoylated CaN, we see less pink AuNP solutions, visually indicating AuNP aggregation, whereas N_3_-CaN-clicked particles have a deep pink color, indicating no aggregation. **B** The activity of calmodulin conjugated nanoparticles is tested with a calcineurin phosphatase activity assay. Three controls (bare, alkyne, WT-CaM) represent nanoparticles with no expected covalent protein surface modification. N_3_-CaM represents the experimental group where CaM with the 12-ADA tag was incubated with alkyne-functionalized gold nanoparticles. (**p < 0.01, *n* = 3)

Next, we tested the stability of the 100-nm AuNPs after CuAAC-mediated conjugation to N_3_-CaN. The control was AuNP samples that underwent CuAAC with Myr-CaN, which does not contain an azide group and therefore should not click to the alkyne on the AuNP surface. We observed that under CuAAC conditions, with proteins lacking the 12-ADA tag, the AuNPs exhibit instability and therefore aggregation effects. For example, in Fig. 6A, the vials containing Myr-CaN AuNP control group exhibited a less pink colored solution than the particle vials containing the N_3_-CaN. This change in color indicates that aggregation occurred in the Myr-CaN vial, due to plasmonic effects [67]. On the other hand, when AuNPs underwent CuAAC in the presence of N_3_-CaN, the resulting solution is deeper pink in color, indicating little aggregation (Fig. 6A, right panel). This is a quick visual confirmation to determine that the protein has a 12-ADA tag and has been successfully “clicked” to the AuNP surface.

### Activity assay of N_3_-CaM-functionalized AuNP

Next, we measured the ability of azide-labeled and particle-conjugated proteins to maintain their function. The activity of N_3_-CaM-conjugated to the AuNP surface with CuAAC was determined indirectly with a CaN phosphatase assay. Increasing concentrations of phosphate (PO_4_^3−^) in the solution indicate increasing levels of CaN phosphatase activity, as CaN dephosphorylates a peptide substrate when it is held in its active state with CaM in the presence of Ca^2+^ [52]. AuNPs were functionalized with alkyne-PEG4-amine and conjugated to N_3_-CaM particles (N_3_-CaM). As controls, we used non-functionalized AuNPs (bare), alkyne-PEG4-amine-coated AuNPs (alkyne), and alkyne-coated AuNPs that underwent CuAAC conditions using wildtype calmodulin (WT-CaM). We found, with statistical significance (** *p* < 0.01), that the N_3_-CaM remains active post-conjugation to the nanoparticles (Fig. 6B). The control groups, however, demonstrate low levels of PO_4_^3−^ present after incubation with Ca^2+^ and CaN. From this result, we conclude that the CuAAC conjugation method onto the AuNPs does not inhibit N_3_-CaM activity. Further, this result demonstrates the accessibility of N_3_-CaM on the particle surface to interact with the free CaN. This is promising for future applications, such as biosensor design, where bio-active proteins must be accessible to binding partners to produce measurable signal output [31].

### Conjugation of protein kinase A (PKA) to magnetic particles

**Fig. 7:**
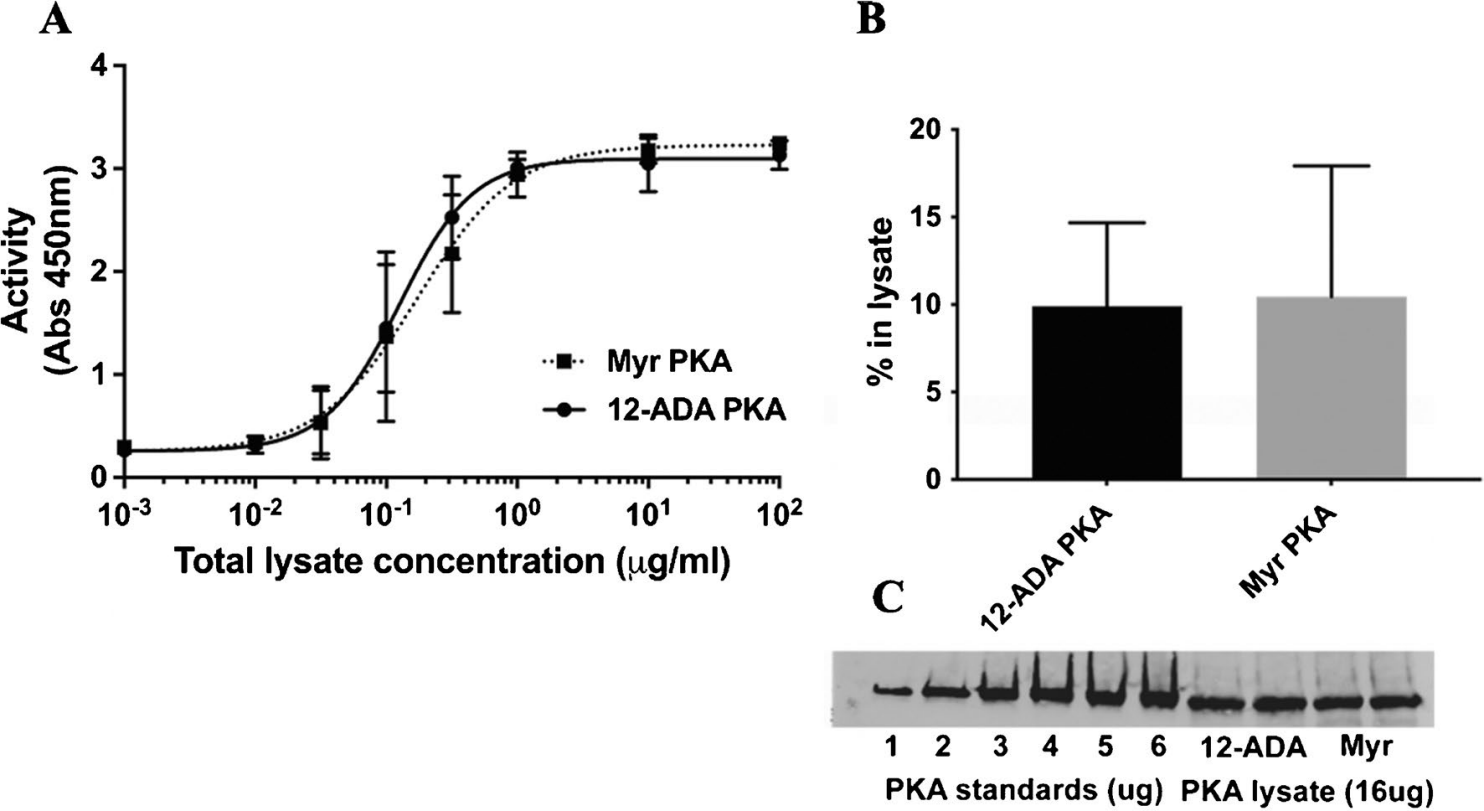
Activity of 12-ADA and myristic acid-tagged PKA expressed in vivo. **A** PKA was expressed in BL21 *E. coli* cells in the presence of 12-ADA or myristic acid. Activities of both variants, measured at different protein concentrations, were plotted. Both variants of PKA showed similar activity (*n* = 3 repeats, 2 replicates for each repeat). Error bars are represent standard deviation. **B** Similar levels of 12-ADA PKA and Myr PKA in lysates were obtained using quantitative western blotting (*n* = 3). **C** Representative western blot used for quantification in **B**

Protein kinase A, also known as cAMP-dependent kinase or PKA, is an important serine-threonine kinase that regulates a number of cellular functions including molecular regulation of neuronal synaptic connections, glucose and lipid metabolism, and contractility in cardiac myocytes, and more [68] PKA is naturally myristoylated and easy to express in *E. coli*; thus, it is readily suited for recombinant expression and tagging with 12-ADA, similar to our previously published methods [35]. To test if the activity of PKA is affected by the addition of the 12-ADA tag, we compared the activities of both 12-ADA-tagged PKA (12-ADA PKA) and myristic acid-tagged PKA (Myr PKA) (Fig. 7A). Activities of both variants of PKA were measured at varying concentration of the lysate in which they were present. As illustrated in Fig. 7, appending 12-ADA to PKA does not markedly affect the activity. Furthermore, using quantitative western blotting, we verified that the percentage of 12-ADA and myristic acid-tagged PKA was similar in their respective lysates (Fig. 7B–C).

### Activity of PKA attached to magnetic beads

**Fig. 8:**
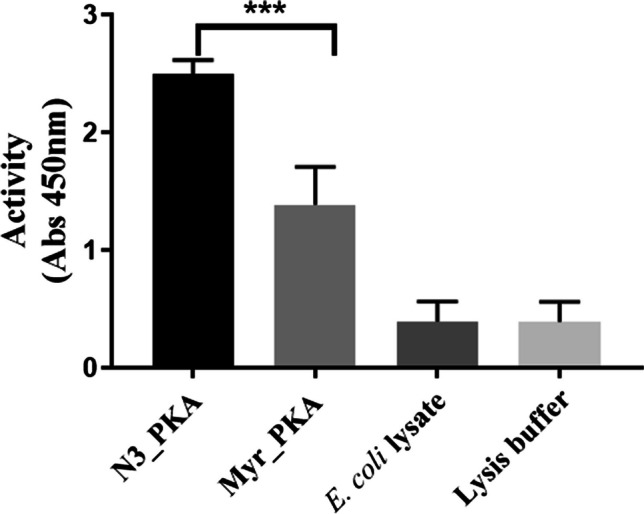
PKA remains active when it is bound to beads: functionalized magnetic beads incubated with cell lysates containing 12-ADA PKA show significantly higher activity compared to beads incubated with Myr PKA (*p* = 0.0002, unpaired *t*-test). Control beads incubated with lysate with no overexpressed PKA and buffer with no protein showed significantly less activity (*n* = 3 repeats, 2 replicates per each repeat)

Next, we tested whether 12-ADA PKA maintained activity upon conjugation to magnetic beads. 12-ADA PKA expressed in clarified *E. coli* lysate was conjugated to magnetic beads functionalized with DBCO as described in “Methods.” An ELISA-based PKA kinase activity kit was used to measure the ability of PKA to phosphorylate a PKA substrate. *E. coli* lysate with no overexpressed PKA showed similar level of activity as that of buffer with no protein, confirming that the lysate contains no proteins that can phosphorylate substrates of PKA. Some kinase activity was seen in controls with *E. coli* lysate containing myristic acid-labeled PKA (Myr_PKA), likely due to non-specific surface interactions. About a 1.6-fold more PKA activity was measured with magnetic beads that were conjugated in *E. coli* lysate containing overexpressed 12-ADA-labeled PKA (N3_PKA) relative to Myr_PKA (Fig. 8). This confirms higher surface labeling of beads with PKA, when covalent labeling is used via click chemistry. Furthermore, the azide tag and covalent labeling do not inhibit the activity of PKA.

### Functional assays with PKA-tagged magnetic beads

**Fig. 9:**
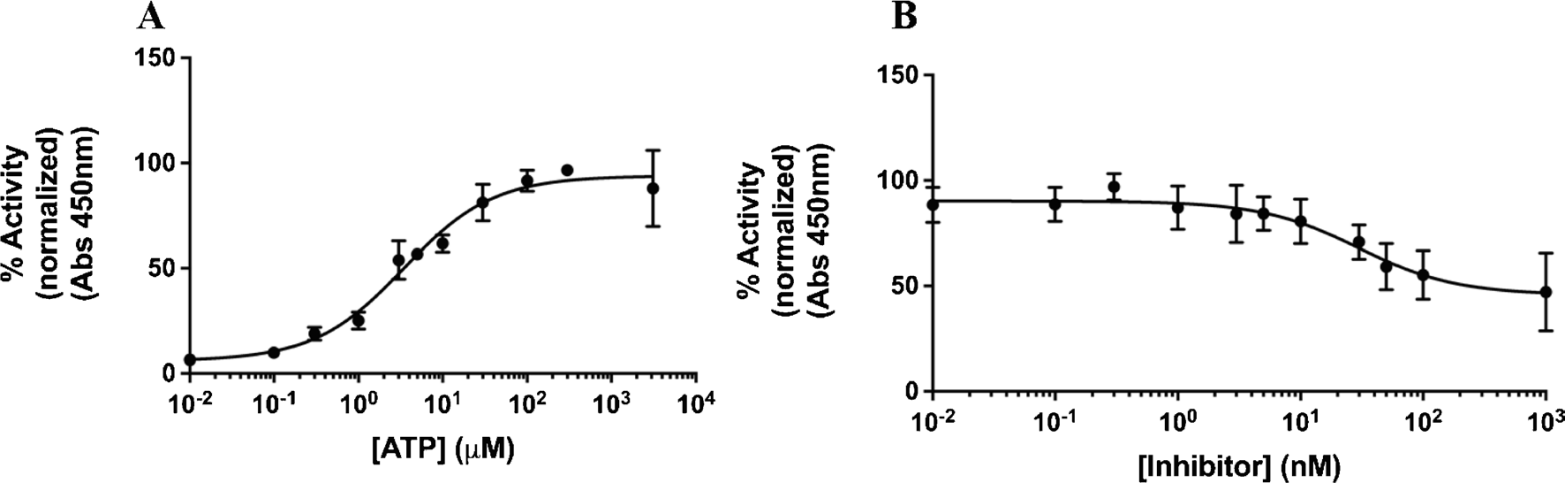
Activity of PKA attached to magnetic beads at varying concentrations of ATP and inhibitor. **A** Level of phosphorylation of a substrate by PKA attached to magnetic beads is plotted as a function of ATP concentration. The obtained dissociation constant of 3.4μM for ATP unbinding from PKA (*n* = 3 repeats, 3 replicates for each repeat). **B** Level of phosphorylation of a substrate by PKA attached to magnetic beads is plotted as a function of inhibitor concentration (*n* = 3 repeats, 2 replicates for each repeat). GraphPad Prism was used to fit the data (nonlinear fit of data variable slope four parameters). Error bars represent standard deviation

Having confirmed that 12-ADA PKA remains active upon conjugation to magnetic beads (12-ADA PKA beads), we performed functional assays to determine if activity is maintained at wildtype (WT) levels. We measured the activity of 12-ADA PKA beads in the presence of varying concentrations of ATP and an inhibitor of PKA (Protein Kinase A Inhibitor Fragment 6-22 amide). The concentration of ATP that gave 50% maximal activity (EC_50_) was 3.4±0.4 μM (Fig. 9A). This is reasonably close to the value obtained using purified PKA (7.7 μM) by other researchers [69]. Further characterization of the 12-ADA PKA beads showed that the IC_50_ value for the PKA inhibitor obtained is (29.8±10.4 nM) (Fig. 9B), which is higher than the IC_50_ previously shown using purified PKA (1.6 nM) [70]. This difference in IC_50_ values could be because of the different buffers used in their experiments and the usage of (γ-^32^P] ATP instead of ATP. PKA is known to show different phosphorylation efficiencies in the presence of different analogs of ATP [71]. Taken together, these results suggest that PKA retains functional characteristics when conjugated to beads and that this assay, where PKA is directly conjugated from lysate without prior purification, is amenable for high-throughput activity measurements.

While the protein conjugations methods developed in this work have been shown to be specific to the proteins of interest, a limitation of the current work are that only recombinantly expressed and 12-ADA-tagged proteins are targeted for conjugation. There exist few methods that can target a single protein of interest directly from cell lysate without some recombinant expression, genetic modification, or tagging. These methods, however, join the ever-growing list of technologies that allow for selective tagging and conjugation. Other limitation is that recombinant expression in this system is limited to prokaryotic expression systems. In eukaryotic expression systems, naturally myristoylated proteins would also be labeled with the 12-ADA tag. There is a high potential for cell-free expressions systems to overcome this limitation [72].

## Conclusion

We report here the ability to functionalize gold nanoparticles and magnetic beads with bio-active 12-ADA-labeled purified proteins or directly from clarified cell lysates. We believe that these approaches will find utility in a variety of biotechnology applications, such as protein–protein binding kinetics, nanoparticle-based drug delivery systems, bio-active materials development, and *in situ* protein-based diagnostic approaches. Conjugating engineered proteins to AuNPs directly from cell lysates would significantly streamline laboratory and manufacturing workflows by reducing the number of steps needed to produce protein-conjugated particles. Protein purification often needs to be tailored to the protein that is purified and often takes hours if not days to complete. Eliminating this step altogether significantly saves time and material costs. Another advantage of conjugating to particles directly from cell lysate is that the protein can be kept in a native cellular environment and immediately conjugated after lysis. The need for expensive protease inhibitors, harsh purification conditions, or storage at ultra-low temperature in buffered solutions is not necessary. Engineering recombinant proteins functionalized with click chemistry enabled tags, and the facile conjugation of those proteins to click chemistry-functionalized nanoparticles can be combined in any number of ways to produce protein–solid surface conjugates with desired properties.

Many chemoenzymatic tagging methods [37] and site selective incorporation of non-canonical amino acids also allow for proteins to be functionalized with click chemistry functionality in a protein-specific and residue-specific manner [38, 39]. Together with the work presented here showing that click chemistry-functionalized particles remain monodisperse and functional for at least 9 months, the methods we present for rapid conjugation to click chemistry-functionalized proteins will be a highly advantageous technique for future research and protein–nanoparticle development in a number of fields, especially when bio-active conjugates are desired. Limitations of the reported protein conjugation methods are that only recombinantly expressed proteins can be selectively tagged with 12-ADA, and that protein selective protein tagging with 12-ADA is confined to prokaryotic expression systems.

### Supplementary information

ESM 1 (PDF 636 kb)
