# Supplementary material for: Immobilization of azide-functionalized proteins to micro- and nanoparticles directly from cell lysate
Source: Mikrochim Acta. 2023 Dec 22;191(1):46. doi: 10.1007/s00604-023-06068-4 (PMC10739308; doi:10.1007/s00604-023-06068-4)
Supplement: Supplementary file 1 — (PDF 636 kb) [file 604_2023_6068_MOESM1_ESM.pdf]

Electronic Supplementary Information

Immobilization of azide-functionalized proteins to micro and nanoparticles directly from cell lysate

Gunjan Saini[1§], Mrugesh Krishna Parasa[1§], Katherine N. Clayton[2§], Julia G. Fraseur[1§], Scott C. Bolton[1], Kevin P. Lin[3], Steven T. Wereley[2], Tamara L. Kinzer-Ursem[1*]

[1]Weldon School of Biomedical Engineering, Purdue University, West Lafayette, Indiana, 47906,
[2]School of Mechanical Engineering, Purdue University, West Lafayette, Indiana 47906, USA
[3]Department of Biochemistry, Purdue University, West Lafayette, Indiana 47906, USA

§Authors contributed equally to this work
*Corresponding author email: tursem@purdue.edu

Synthesis of 12-ADA

A mixture of 12-bromododecanoic acid (1.8 g, 0.0064 mol), sodium azide (1.2 g, 0.019 mol), and 18-crown-6 (0.5 g, 0.0019 mol) were stirred in 25 mL N, N-dimethylformamide (DMF) under an argon blanket at room temperature overnight. DMF was then removed under vacuum. The residue was diluted with 25 mL dichloromethane, followed by the addition of hydrochloric acid (1 M, 25 mL) to quench unreacted sodium azide. The organic layer was rinsed three times with 25 mL water, dried with Na_2SO_4, and filtered and concentrated under vacuum yielding the desired product (1.5 g, 97% yield) at 95% purity as a pale-yellow liquid. Characterization data for the final product (1H NMR) yielded data that matched published results. 1H NMR 3.19 (t. 2H, J=6.9 Hz, CH2); 2.28 (t. 2H, J=7.5 Hz. CH2): 1.53 (m. 4H, 2xCH2); 1.21 (m. 14H) plus 18-crown-6 as a residual impurity 1H NMR 3.51(s. 2H, CH2).

Methods of Fluorescent Protein Labeling

The entire reaction was performed in a 1.5 mL Eppendorf micro-centrifuge tube containing: 30 µL of azide-labeled protein (pure or in cell lysate) and 30 µL ultrapure water, 10 µL of 400 mM sodium ascorbate, 20 µL of 500 mM iodoacetamide, 0.5 µL of 8 mM alkyne-labeled fluorophore (Alexa Fluor 647 for GFP and CaM labeling from Thermo Scientific, Waltham, MA and TAMRA for CaN labeling from Click Chemistry Tools, Scottsdale, AZ), 16 µL of 25 mM $CuSO_4$, 40 µL of 50 mM THPTA, and 40 µL of 100 mM aminoguanidine. Contents were incubated on a rotator for 30 minutes at room temperature to ensure complete reaction. Precipitation of fluorescently-labeled protein was achieved by an initial round of protein-fluorophore incubation with 1 mL of cold acetone for 20 minutes at -20°C, followed by centrifugation (10,000 x g, 10 minutes). The protein

solution was again mixed with 1 mL of cold acetone and centrifuged (10,000 x g, 10 minutes). The protein pellet formed via centrifugation was air-dried at -20°C and resuspended in 1X Laemmli buffer with 5% BME. Protein concentration was determined with a Pierce™ 660 Protein Assay with Ionic Detergent Compatibility Reagent (IDCR) and the sample was further analyzed with SDS-PAGE (4-20% Mini-PROTEAN® TGX™ Precast Protein Gels, 10-well, 120 V for 110 minutes) to determine protein molecular weight, azide labeling, and protein sample purity. Fluorescent gels were imaged with the Azure system. Further, the SDS-PAGE gel was stained with Coomassie (GelCode Blue, Thermo Fisher Scientific, Erie, NY) for 30 minutes and destained in deionized water overnight. The Coomassie stained SDS-PAGE gel was imaged with Odyssey CLx software (LI-COR, Lincoln, NE).

Table S1. DLS size and PdI values for NHS-DBCO AuNPs and Streptavidin-DBCO AuNPs.

	**NHS-DBCO**	**Streptavidin-DBCO**
**Size (nm)**	326.6±72.3	142.1±1.5
**PdI**	0.62±0.12	0.26±0.01

Table S2. DLS size values for Streptavidin-DBCO click conjugated N3-CaM AuNPs.

	**Streptavidin**	**DBCO**	**WT-CaM**	**N3-CaM**	**$CaCl_2$ N3-CaM**	**EGTA N3-CaM**
**Size (nm)**	129.8±0.4	130.2±1.5	130.4±0.6	132.8±0.6	132.9±0.6	134.0±1.4

Table S3. DLS size and PdI values for NHS-Alkyne AuNPs.

	**New**	**9 months**
**Size (nm)**	134.3±1.9	131.2±0.4
**PdI**	0.092±0.006	0.025±0.008

Table S4. Activity assay for CaM clicked AuNPs.

	**Bare**	**Alkyne**	**WT-CaM**	**N3-CaM**
**PO_4 (nmol)**	0.031±0.019	0.030±0.010	0.109±0.151	0.489±0.123

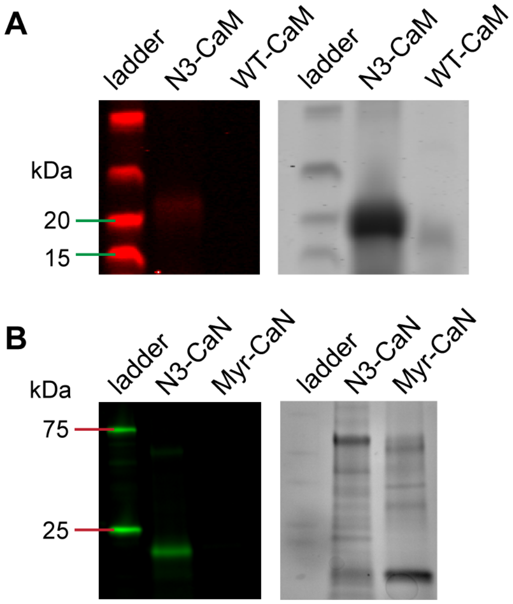

Figure S1. Click gel verification of (A) pure N_3-CaM, and WT-CaM and (B) pure N_3-CaN, Myr-CaN.

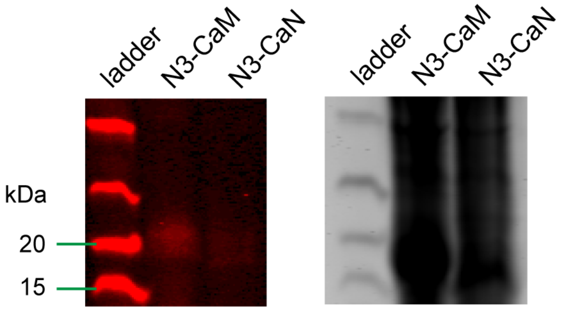

Figure S2. Click gel verification of N_3-CaN and N_3-CaM from lysates.

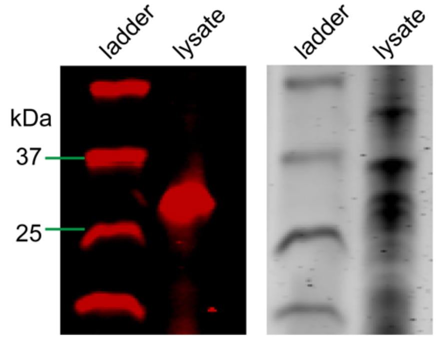

Figure S3. Click verification gel of N_3 GFP. The only fluorescing band appears between 25 and 37 kDa (emGFP MW = 27 kDa). Coomassie stain of the same gel shows multiple banding patterns, indicative of cell lysates.

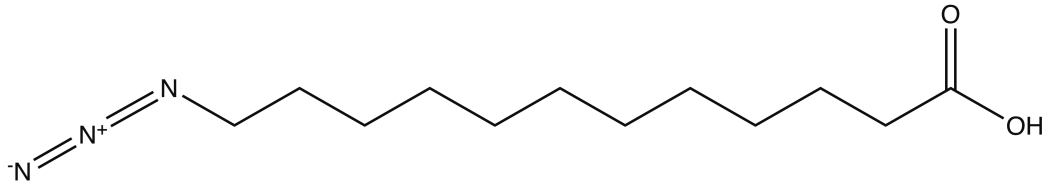

Figure S4. Structure of 12-ADA

	**DBCO-amine**	**DBCO-biotin**	**Alkyne-amine**
**Advantages**	Maintain Protein enzymatic functions.	Doesn't affect enzymatic functions	High labeling efficiency and high specificity
**Disadvantages**	Less specific due to absence of catalyst copper	Less specificity due to absence of copper	May adversely affect protein enzymatic function due to excess of copper

Table 1: Showing comparison of three immobilization methods using DBCO-amine, DBCO-biotin, or alkyne-amine. For reviews of copper catalyzed (alkyne) versus strain-promoted (dibenzocyclooctyne and similar reagents) see[1-3]. Dibenzocyclooctynes can have cross reactivity to thiol group of cysteine residues[4,5].

Select reviews articles (there are many others that outline the advantages and disadvantages of these and other click chemistries):

1. Sletten EM, Bertozzi CR. Bioorthogonal chemistry: fishing for selectivity in a sea of functionality. Angew Chem Int Ed Engl. 2009;48(38):6974-98. doi: 10.1002/anie.200900942. PMID: 19714693; PMCID: PMC2864149.

2. Sletten EM, Bertozzi CR. From mechanism to mouse: a tale of two bioorthogonal reactions. Acc Chem Res. 2011 Sep 20;44(9):666-76. doi: 10.1021/ar200148z. Epub 2011 Aug 15. PMID: 21838330; PMCID: PMC3184615.

3. Saleh, A.M., Wilding, K.M., Calve, S. *et al.* Non-canonical amino acid labeling in proteomics and biotechnology. *J Biol Eng* **13**, 43 (2019). https://doi.org/10.1186/s13036-019-0166-3

References related to cyclooctyne reactivity to thiols:

4. van Geel R, Pruijn GJ, van Delft FL, Boelens WC. Preventing thiol-yne addition improves the specificity of strain-promoted azide-alkyne cycloaddition. Bioconjug Chem. 2012 Mar 21;23(3):392-8. doi: 10.1021/bc200365k. Epub 2012 Mar 8. PMID: 22372991. *Authors report methods to reduce undesired thiol-yne reactions.*

5. Zhang C, Dai P, Vinogradov AA, Gates ZP, Pentelute BL. Site-Selective Cysteine-Cyclooctyne Conjugation. Angew Chem Int Ed Engl. 2018 May 28;57(22):6459-6463. doi: 10.1002/anie.201800860. Epub 2018 Apr 26. PMID: 29575377; PMCID: PMC6150453. *Authors take advantage of the thiol reactivity of cyclooctynes to site-selective labeling.*
